# The antiSMASH database version 5

**DOI:** 10.1093/nar/gkaf1210

**Published:** 2025-11-18

**Authors:** Kai Blin, Simon Shaw, Marnix H Medema, Tilmann Weber

**Affiliations:** The Novo Nordisk Foundation Center for Biosustainability, Technical University of Denmark, 2800 Kgs., Lyngby, Denmark; The Novo Nordisk Foundation Center for Biosustainability, Technical University of Denmark, 2800 Kgs., Lyngby, Denmark; Bioinformatics Group, Wageningen University, 6708PB, Wageningen, The Netherlands; The Novo Nordisk Foundation Center for Biosustainability, Technical University of Denmark, 2800 Kgs., Lyngby, Denmark

## Abstract

Specialized metabolites produced by microorganisms are frequently used in the development of drugs and crop protection agents. Genome mining is a widely used approach to access this potential, and antiSMASH is often the tool of choice for this task. Here, we present version 5 of the antiSMASH database, with biosynthetic gene cluster predictions provided by antiSMASH 8.1 available in an easy-to-use web interface. Version 5 of the database contains 833 archaeal, 54 800 bacterial, and 421 fungal genomes and is available from https://antismash-db.secondarymetabolites.org/.

## Graphical Abstract

**Graphical Abstract:**
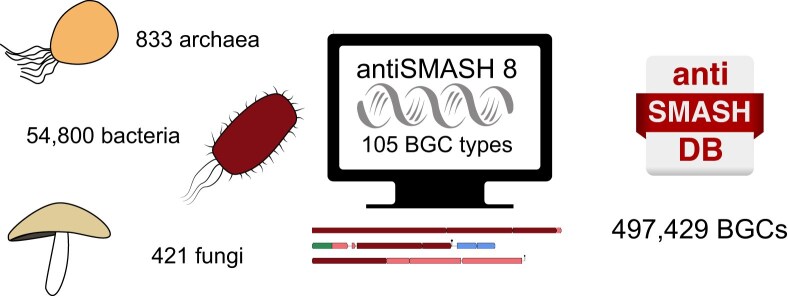

## Introduction

Specialized or secondary metabolites produced by microorganisms are the main source of bioactive compounds that are in use as antimicrobial and anticancer drugs [1], fungicides, herbicides, pesticides, and biostimulants [2]. In the last decades, the increasing availability of microbial genomes has established genome mining as a very important method for the identification of the biosynthetic gene clusters (BGCs) responsible for the synthesis of these compounds [3]. To assist with genome mining, software tools were soon developed, and the field has seen active development in the past decades (see [4–8] for reviews discussing tools and timelines). While there were a number of tools, only a few databases existed to make BGC data available. An example of the latter development is e.g. ClusterMine360 [9], which was introduced in 2013 but has been discontinued in the meantime.

Initially released in 2011, antiSMASH [10–17] has become the most widely applied tool for genome mining for specialized/secondary metabolites and is considered as the gold standard. antiSMASH uses a rule-based system for identifying genome regions containing BGCs based on conserved biosynthetic enzymes. Currently, it detects 105 different pathway types. For some more well-understood synthesis types, it also performs cluster-specific analyses. antiSMASH also compares identified regions to similar regions in the MIBiG database [18] of known BGCs and a dataset of antiSMASH results on publicly available, high-quality genomes.

antiSMASH is designed to annotate and analyse a single genome at a time. To enable answering research questions that require comparative analyses across many genomes, we have developed the antiSMASH database [19–22]. This database provides the foundation of antiSMASH’s ClusterBlast analysis, with results from ClusterBlast linking directly to the database. In addition, the antiSMASH database website provides BLAST-like searches, taxonomic browsing, and an easy-to-use graphical query builder to dynamically search database contents.

Here, we present the fifth version of this database, covering 833 archaeal, 54 800 bacterial, and 421 fungal genomes.

## Materials and methods

### Selection of included genomes

Archaeal, bacterial, and fungal genomes were downloaded from the NCBI RefSeq database [23] on 7 January 2025 using the ncbi-genome-download tool [24] with ‘complete’, ‘chromosome’, and ‘scaffold’ assembly levels, yielding 1267 archaeal, 182 383 bacterial, and 441 fungal assemblies. The quality filtering deviated from previous versions that used the contig count (see [22] for a description). We decided to instead use the L50 value, which is the minimal number of contigs needed to account for half the total assembly length. This decision was made in order to not penalize assemblies that had one or two large contigs but also had a number of small contigs due to repeats, contamination, or leftover adapter sequences. The limits were set to an *L*50 value of at most 10 for archaea and bacteria and 60 for fungi. As described before [22], duplicated genomes were assigned to a similarity cluster when they had a Mash [25] distance of 0.04 or less. Another difference from previous versions is that we selected the representative genome of a similarity cluster using a slightly more complex calculation. Each assembly was assigned a score *S* with $S\; = \;w1\times\;Ln\ - \;w2\times\;L50n$, where *Ln* is the normalized length of all the assemblies in the similarity cluster, *L*50*n* is the normalized *L*50 value of the similarity cluster, *w*1 is a length bonus, and *w*2 is a fragmentation penalty. The assembly in the similarity cluster with the highest score was selected as the representative assembly. For our selection, we used a length bonus and fragmentation penalty of 0.3. This method was chosen because our old contig-count minimization could pick assemblies of genome-minimized, plasmid-free mutants (e.g. *Streptomyces collinus* str. SQ GCF_021496465.1) over their corresponding wild types (e.g. *Streptomyces collinus* str. Tü365 GCF_000444875.1) because the plasmids increased the contig count. After redundancy filtering, 843 archaeal, 55 680 bacterial, and 431 fungal assemblies remained. Of the fungal assemblies, 36 contain genes that carry splice variants outside their coding sequences, leading to CDS entries that have identical locations, locus tags, and translations while having differing protein IDs in the GenBank files. As antiSMASH requires all CDSs to be uniquely identifiable by location, translation, and ID, we removed the duplicate CDSs on those assemblies.

### antiSMASH annotations and data import

With the filtered, non-redundant dataset, we used GNU parallel [26] to run antiSMASH 8 with the options ‘- -cb-knownclusters - -cb-subclusters - -cc-mibig - -rre - -asf’. antiSMASH successfully processed all archaeal, bacterial, and fungal sequences. Using the data from this first run, we extracted all predicted ribosomally synthesized and post-translationally modified peptide (RiPP) precursors and all predicted regions. These were used to build new CompaRiPPson and ClusterBlast datasets, respectively. With these new datasets, antiSMASH 8 was re-run on the first round’s results using the options ‘- -reuse - -cb-general - -clusterhmmer - -tigrfam - -pfam2go’ to allow cross-references to the other results in the antiSMASH database.

The SQL schema for the database and the importer were updated to support antiSMASH 8 results. During the import process, assemblies without any antiSMASH hits were dropped. This results in a final count of 833 archaeal, 54 800 bacterial, and 421 fungal assemblies in the database.

## Results and discussion

**Figure 1.**
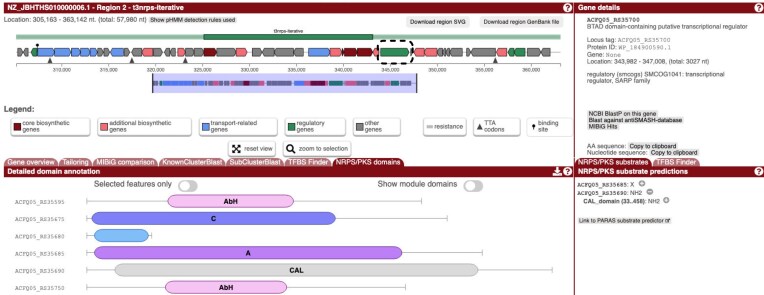
An iterative type III NRPS cluster from *Amycolatopsis umgeniensis* (NCBI assembly ID GCF_042676565.1). The green opaque bar above the gene arrows spans all core genes. A nearby SARP regulator, highlighted by a striped box, strongly suggests that this region indeed contains a BGC.

The NCBI RefSeq database is a valuable resource of a lot of microbial genomic diversity. It does also contain a large number of very similar genomes, especially for common pathogens like *Staphylococcus aureus, Escherichia coli*, or *Salmonella enterica*. It also contains a large number of very fragmented genomes. Fragmentation has been shown to negatively impact BGC detection [27]. To ensure that the antiSMASH database covers the whole microbial tree of life as well as possibly without overly biasing towards the most frequently sequenced organisms, and to ensure the BGC predictions are of the best possible quality, the data were rigorously filtered for quality and sequence dissimilarity. After the filtering and processing, 833 archaeal, 54 800 bacterial, and 421 fungal assemblies are present in the database. Annotations were performed using antiSMASH 8.1, which, on top of the rules described in [17], adds two new detection rules, one for adenosine derivatives like aureonuclemycin and another for iterative NRPS type III clusters (so NRPSs that are thiotemplated but do not use a condensation domain as the peptide-bond forming enzyme, see [28] for a detailed description and Fig. 1 for an example), for a total of 105 supported BGC types.

**Figure 2.**
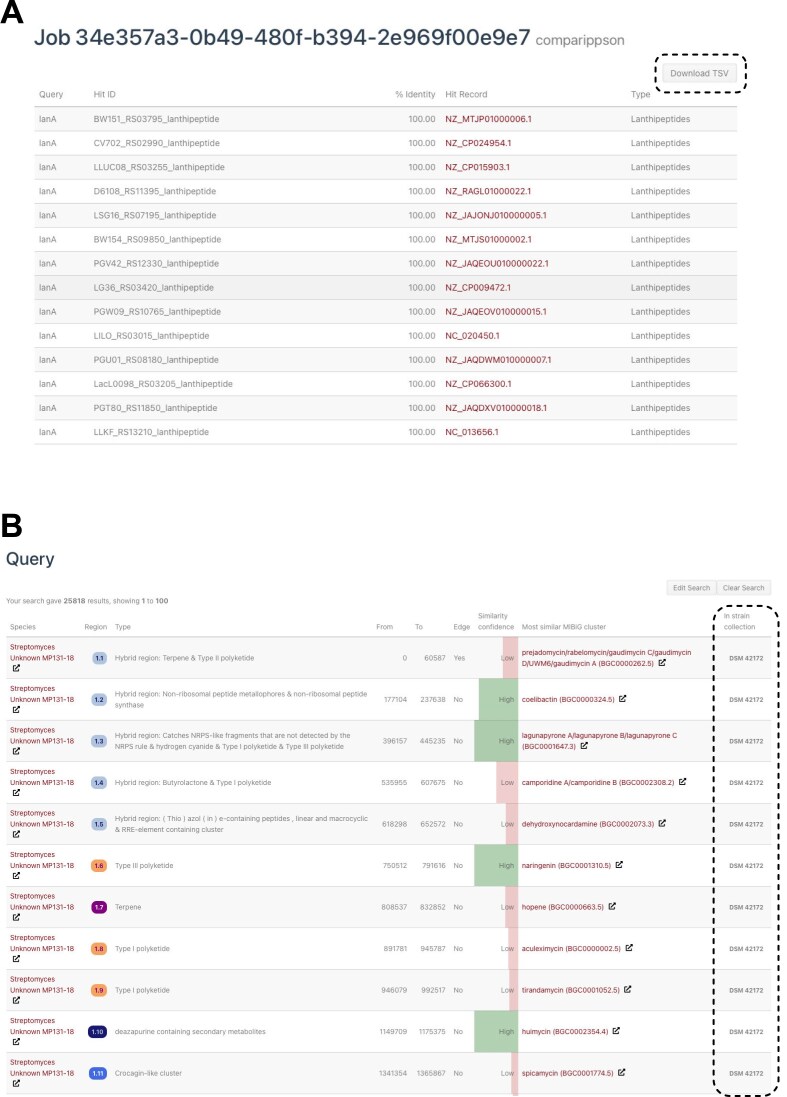
**A**) A screenshot of the RiPP precursor (CompaRiPPson) search results on a nisin-like sequence. The new ‘Download TSV’ button is highlighted with a striped box. (**B**) A screenshot of a ‘genus *Streptomyces* AND available in strain collection’ search. The strain collection identifier column is highlighted with a striped box.

Support for new antiSMASH 8 annotations has been added to the query builder. A common user request for the BLAST-style sequence searches has been to add an option to download the result table. We have now added the option to download the sequence search results table (Fig. 2A). To make it easier for researchers to identify strains they can obtain from strain collections, we have worked with colleagues from the German Collection of Microorganisms and Cell Cultures (DSMZ) and the DTU Biosustain NBC strain collection to flag strains that are available to order (Fig. 2B). We are in contact with other strain collections about getting their strains included in a future release.

Compared to version 4 with 36 554 assemblies containing 231 534 BGC regions not touching a contig edge, version 5 raises these numbers to 56 054 assemblies (a 53% increase) and 497 429 BGC regions (a 107% increase). The CompaRiPPson dataset of predicted RiPP precursor peptides has grown from 16 533 to 34 401 sequences (a 108% increase).

## Conclusion

Genome mining continues to be a valuable tool for assessing microbial biosynthetic potential. In the past 15 years, antiSMASH has aided these efforts. The antiSMASH database provides a user-friendly web interface to compare, contextualize, and cross-reference findings across genomes. With 497 429 BGC regions across archaea, bacteria, and fungi, the antiSMASH database version 5 is a comprehensive collection of specialized/secondary metabolite biosynthetic gene clusters with high-quality, up-to-date antiSMASH annotations for the whole natural product research community.

## Data Availability

The antiSMASH database is available at https://antismash-db.secondarymetabolites.org/. There are no access restrictions for academic or commercial use of the web server. The source code components and SQL schema for the antiSMASH database are available on GitHub (https://github.com/antismash) under an OSI-approved Open Source license. The complete set of antiSMASH results, antiSMASH JSON files, and an SQL dump of the database can be downloaded from the antiSMASH download server (https://dl.secondarymetabolites.org/database/5.0).
